# Atractylodin Induces Apoptosis and Inhibits the Migration of A549 Lung Cancer Cells by Regulating ROS-Mediated Signaling Pathways

**DOI:** 10.3390/molecules27092946

**Published:** 2022-05-05

**Authors:** Tong Zhang, Shu-Mei Li, Yan-Nan Li, Jing-Long Cao, Hui Xue, Chang Wang, Cheng-Hao Jin

**Affiliations:** 1 College of Life Science & Technology, Heilongjiang Bayi Agricultural University, Daqing 163319, China; zhangtong@byau.edu.cn (T.Z.); lyn2284593531@163.com (Y.-N.L.); long91812022@163.com (J.-L.C.); xuehui22712022@163.com (H.X.); 2 Hemodialysis Center, Daqing Oilfield General Hospital, Daqing 163001, China; lishumei19751202@163.com; 3 College of Science, Heilongjiang Bayi Agricultural University, Daqing 163319, China; 4 National Coarse Cereals Engineering Research Center, Daqing 163319, China; 5 College of Food Science & Technology, Heilongjiang Bayi Agricultural University, Daqing 163319, China

**Keywords:** Atractylodin, lung cancer, cell apoptosis, cell migration, cell cycle, reactive oxygen species

## Abstract

Atractylodin (ATR) has anticancer effects on some tumor cells by inducing apoptosis, but its mechanism in lung cancer remains unclear. This study investigates the inhibitory effect of ATR on A549 lung cancer cells. Cell viability was detected by the Cell Counting Kit-8 assay, and results showed that ATR could significantly inhibit the proliferation of A549 cells. Apoptosis was detected by Annexin V-FITC/PI staining, and apoptosis rate and mitochondrial membrane potential were detected by flow cytometry. Results showed that the effect of ATR on the apoptosis of A549 cells was negatively correlated with the change in mitochondrial membrane potential. Western blot analysis showed that ATR regulated apoptosis induced by mitogen-activated protein kinase, signal transducer and activator of transcription 3, and nuclear factor kappa B signaling pathways. Analyses of reactive oxygen species (ROS), cell cycle, and cell migration showed that ATR induced intracellular ROS accumulation as an initiation signal to induce cell cycle arrest regulated by the AKT signaling pathway and cell migration inhibition regulated by the Wnt signaling pathway. Results showed that ATR can inhibit cell proliferation, induce cell apoptosis, induce cell cycle arrest, and inhibit the migration of A549 cells (*p* < 0.05 was considered statistically significant, * *p* < 0.05, ** *p* < 0.01 and *** *p* < 0.001).

## 1. Introduction

Lung cancer is a malignant tumor that originates from the bronchial mucosa, gland, or alveolar epithelium [1]. Lung cancer incidence and mortality are the highest in industrially developed countries or cities [2]. Lung cancer cells are characterized by rapid proliferation and prone to distant metastasis [3,4,5]. Despite great advances in the treatment of lung cancer, its prognosis remains poor, with a 5-year survival rate of less than 20% in patients treated with radiotherapy [6]. Therefore, it is urgent to find effective drugs for this disease.

Inducing the apoptosis of cancer cells is an effective way to treat cancer, and many signaling pathways are involved in this [7]. A large number of anticancer drugs exert antitumor activity by regulating the body’s oxidative stress response [8,9,10]. High levels of reactive oxygen species (ROS), as highly active molecules, regulate many signaling pathways. Mitogen-activated protein kinase (MAPK) is activated by extracellular stimuli and mediates signal transduction from cell membrane to nucleus, which regulates many physiological activities [11]. The signal transducer and activator of the transcription 3 (STAT3) signaling pathway plays a key role in the regulation of apoptosis and pathogen resistance [12]. Some of its phosphorylation sites form proteins that link it to MAPK, AKT, and nuclear factor kappa B (NF-κB) signaling pathways. The STAT3 and NF-κB signaling pathways are both downstream of the MAPK signaling pathway and jointly induce apoptosis [13,14,15].

The breakdown of adhesion between cells promotes the migration and invasion of cancer cells [16]. The activation of the Wnt signaling pathway occurs in a variety of tumor models [17]. The Wnt signaling pathway induces epithelial–mesenchymal transition conversion by inhibiting glycogen synthase kinase-3β (GSK-3β)-mediated phosphorylation and β-catenin degradation in the cytoplasm [18,19,20]. β-Catenin also forms complexes with E-cadherin and is located on cell membranes, playing an important role in maintaining intercellular adhesion [21]. Therefore, the inhibition of the Wnt signaling pathway as a novel approach for anticancer therapeutics is a research hotspot.

Natural drugs are popular in all fields of research and development because of their high efficiency and low toxicity [22,23,24]. Atractylodin (ATR) is the main active ingredient with the highest content in *Atractylodes lancea*, and its hypoglycemic, anti-inflammatory, and gastric empty-promoting effects were widely reported [25,26,27]. Some in vivo studies showed that ATR can increase heart rate and decrease diastolic blood pressure in rats, thereby exerting a positive inotropic effect [28]. In addition, the activity of ATR was analyzed by using an in vivo zebrafish model, and ATR had potential antiangiogenic activity [29]. ATR also has good inhibitory effects on malignant tumors such as cholangiocarcinoma and colon cancer [30,31]; however, molecular mechanisms underlying the effects of ATR on lung cancer have not been extensively studied.

This study investigated the mechanism by which ATR inhibits growth and migration, and induces the apoptosis of A549 lung cancer cells. The mechanism of action of ATR in A549 cells, and the relationship between ROS accumulations induced by ATR in A549 cells was discussed.

## 2. Results

### 2.1. Effects of ATR on Cytotoxic Activity

**Figure 1:**
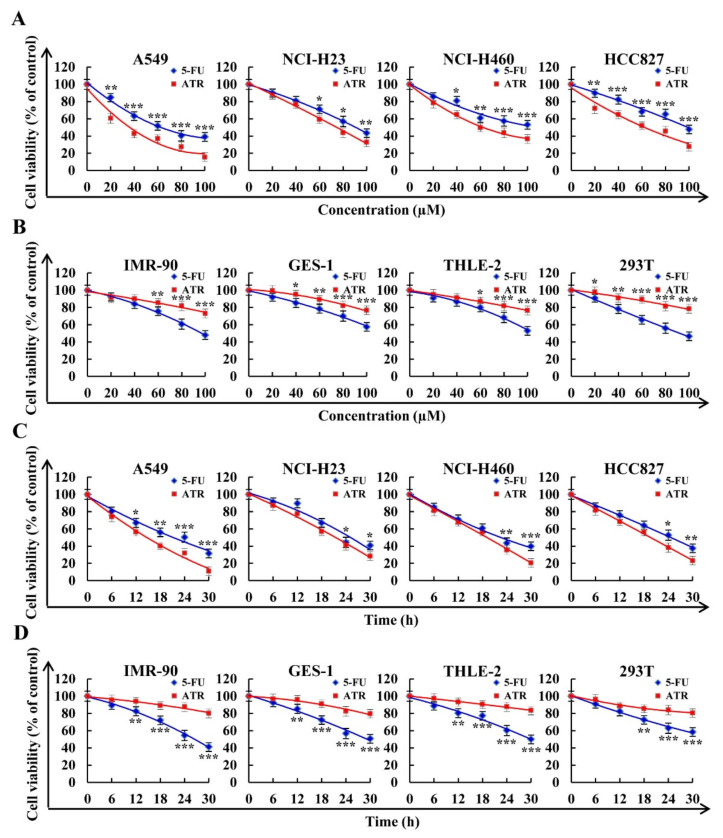
Cytotoxic effects of 5-FU and ATR on cells. Cells treated with different doses of fluorouracil (5-FU) and ATR (20, 40, 60, 80, and 100 µM) for 24 h, and cell viability was detected by CCK-8 assay. (**A**) Viability of human lung cancer cell lines (A549, NCI-H23, NCI-H460, and HCC827). (**B**) Viability of human normal cell lines (IMR-90, GES-1, THLE-2, and 293T). Cells treated with 5-FU and ATR for 6, 12, 18, 24, and 30 h, and cell viability was detected by CCK-8 assay. (**C**) Viability of human lung cancer cell lines (A549, NCI-H23, NCI-H460, and HCC827). (**D**) Viability of human normal cell lines (IMR-90, GES-1, THLE-2, and 293T). * *p* < 0.05, ** *p* < 0.01 and *** *p* < 0.001 vs. 5-FU group.

ATR inhibited the proliferation of lung cancer cells in concentration- and time-dependent manners. As shown in Figure 1A,C, ATR showed good cytotoxicity in the A549, NCI-H23, NCI-H460, and HCC827 lung cancer cell lines. Concentrations that caused 50% cell growth inhibition (IC_50_) values of the four lung cancer cells were 37.92 ± 1.59, 76.88 ± 2.21, 63.27 ± 1.48, and 61.05 ± 1.66 µM (Table 1). In addition, ATR showed low cytotoxicity in the four normal cell lines of IMR-90, GES-1, THLE-2, and 293T (Figure 1B,D). Since A549 cells had the lowest IC_50_ value and were more sensitive to ATR, A549 cells were used for subsequent experiments.

### 2.2. ATR Induces A549 Cell Apoptosis

**Figure 2:**
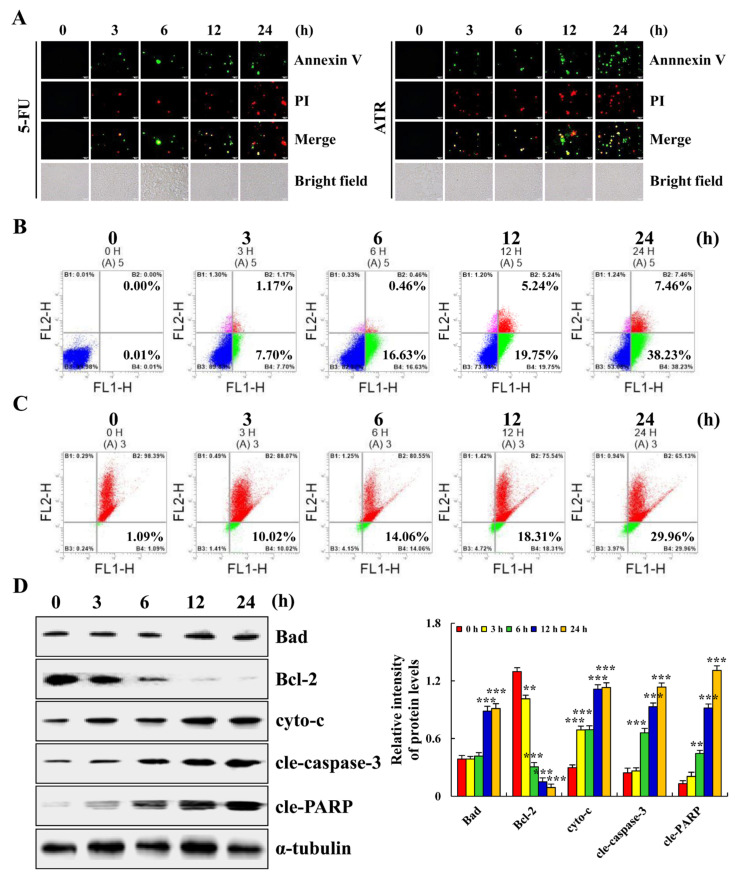
Apoptotic effects of ATR on A549 cells. A549 cells treated with 38 µM ATR for 3, 6, 12, and 24 h. (**A**) Annexin V-FITC/PI staining and fluorescence microscopy images (original magnification, 200×). (**B**) Annexin V-FITC/PI staining and flow cytometry analysis of apoptosis. (**C**) JC-1 staining and flow cytometry analysis of MMP. (**D**) Expression levels of apoptosis-related proteins detected by Western blot analysis with α-tubulin as internal reference. * *p* < 0.05, ** *p* < 0.01 and *** *p* < 0.001 vs. 0 h.

ATR induced the apoptosis of A549 cells. As shown in Figure 2A, compared with ATR treated for 0 h, A549 cells treated with ATR for 24 h showed obvious apoptotic morphology. At the same time, the amount of apoptosis of A549 cells treated with ATR for 24 h was more than that of control drug 5-FU for 24 h. In addition, compared with the untreated group, the apoptosis rate of A549 cells increased by 45.68% after 24 h of ATR treatment, while the level of mitochondrial membrane potential (MMP) of A549 cells decreased by 28.87% (Figure 2B,C). ATR induced the apoptosis of A549 cells by damaging mitochondria. Subsequently, the expression levels of proapoptotic proteins Bad, cyto-c, cle-caspase-3 and cle-PARP were increased, while the expression levels of antiapoptotic protein Bcl-2 were decreased, further confirming the above conclusion (Figure 2D).

### 2.3. ATR Mediates MAPK, STAT3, and NF-κB Signaling Pathways to Induce A549 Cell Apoptosis

**Figure 3:**
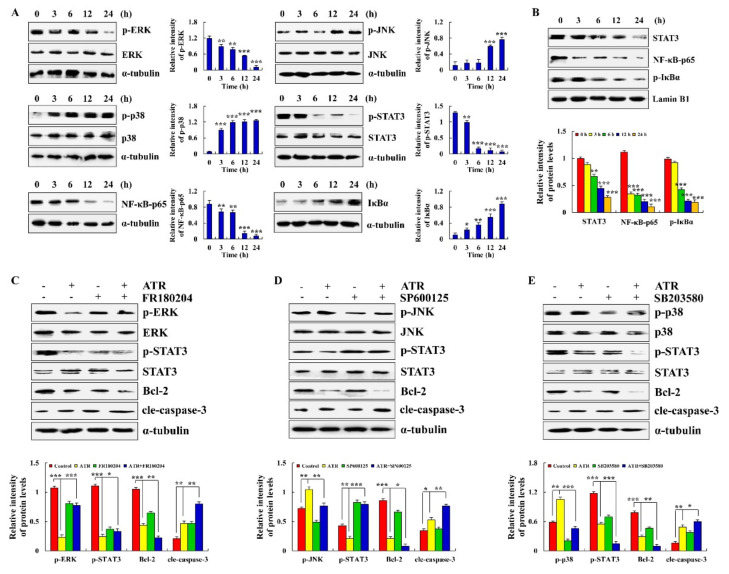
Effects of ATR on MAPK, STAT3, and NF-κB signaling pathways. A549 cells treated with 38 µM ATR for 3, 6, 12, and 24 h. (**A**) Expression levels of MAPK, STAT3, and NF-κB signaling pathway-related proteins detected by Western blot analysis. (**B**) Expression levels of nuclear proteins STAT3, NF-κB-p65, and p-IκBα. * *p* < 0.05, ** *p* < 0.01 and *** *p* < 0.001 vs. 0 h. A549 cells treated with 38 µM ATR and/or 10 µM MAPK signaling pathway inhibitors for 24 h. (**C**) Expression levels of p-ERK, p-STAT3, Bcl-2, and cle-caspase-3. (**D**) Expression levels of p-JNK, p-STAT3, Bcl-2, and cle-caspase-3. (**E**) Expression levels of p-p38, p-STAT3, Bcl-2, and cle-caspase-3. Lamin B1 and α-tubulin used as internal references. ** *p* < 0.01 and *** *p* < 0.001 vs. control or ATR + MAPK inhibitor groups.

ATR induces the apoptosis of A459 cells by regulating the MAPK, STAT3, and NF-κB signaling pathways. Compared with the untreated groups, the expression levels of p-JNK, p-p38 and IκBα increased in the ATR-treated groups, whereas the expression levels of p-ERK, p-STAT3, and NF-κB-p65 decreased (Figure 3A). The protein expression levels of nuclear proteins STAT3, NF-κB-p65, and p-IκBα all showed a downward trend (Figure 3B). Cells were further pretreated with MAPK pathway inhibitors. As shown in Figure 3C–E, an ERK inhibitor (FR180204) and p38 inhibitor (SB203580) reduced the expression levels of p-ERK and p-p38, and of p-STAT3. A JNK inhibitor (SP600125) decreased the expression level of p-JNK, but increased the expression level of p-STAT3.

### 2.4. ATR Regulates MAPK, STAT3, and NF-κB Signaling Pathways by Upregulating ROS

**Figure 4:**
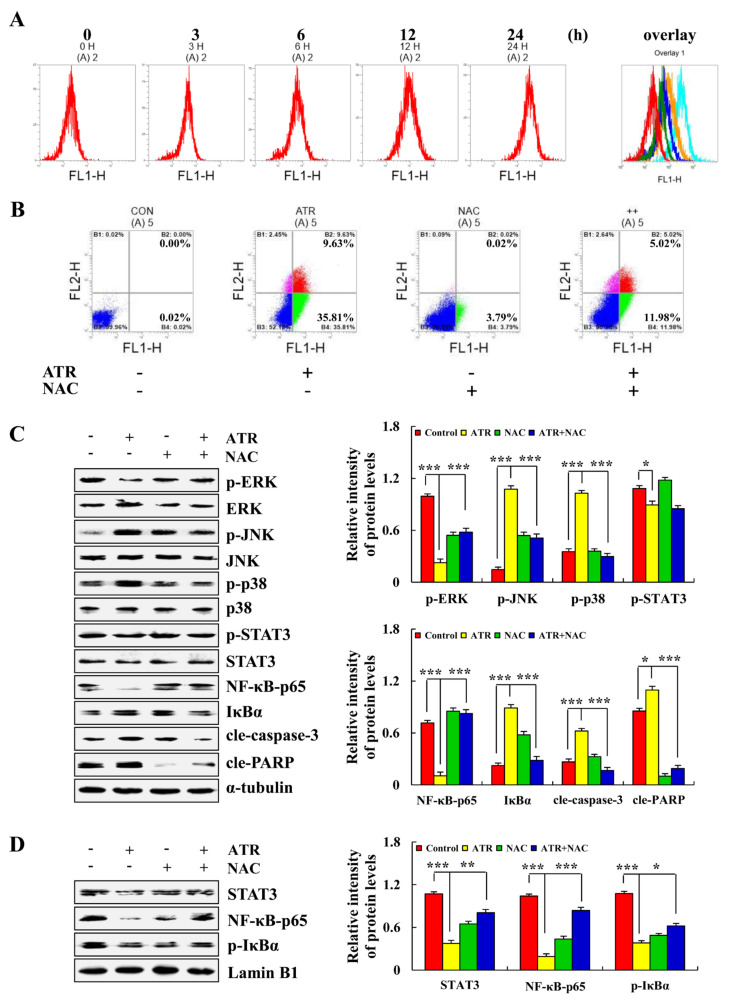
Effects of ATR on ROS levels. A549 cells treated with 38 µM ATR for 3, 6, 12, and 24 h. (**A**) DCFH-DA staining and flow cytometry analysis of ROS levels. A549 cells treated with 38 µM ATR and/or 10 mM NAC for 24 h. (**B**) Annexin V-FITC/PI staining and flow cytometry analysis of apoptosis. (**C**) Expression levels of MAPK, STAT3, and NF-κB signaling pathway-related proteins, cle-caspase-3, and cle-PARP proteins detected by Western blot analysis. (**D**) Expression of nuclear-related proteins detected by Western blot analysis. Lamin B1 and α-tubulin used as internal references. * *p* < 0.05, ** *p* < 0.01 and *** *p* < 0.001 vs. control or ATR + NAC groups.

ATR induced the apoptosis of A549 cells by regulating ROS accumulation. As shown in Figure 4A, ROS levels in A549 cells gradually increased with the extension of ATR treatment time. In order to further verify the relationship between ATR-induced apoptosis of A549 cells and ROS accumulation, reactive oxygen scavengers N-acetyl cysteine (NAC) were added to A549 cells for pretreatment before ATR treatment. Results showed that the addition of NAC reduced the apoptosis rate of A549 cells from 45.44% to 17%, and significantly inhibited apoptosis induced by ATR (Figure 4B). In addition, NAC inhibited the expression level of apoptotic proteins after ATR treatment, and increased the expression level of nuclear proteins in A549 cells after ATR treatment (Figure 4C,D).

### 2.5. ATR Arrests A549 Cell Cycle

**Figure 5:**
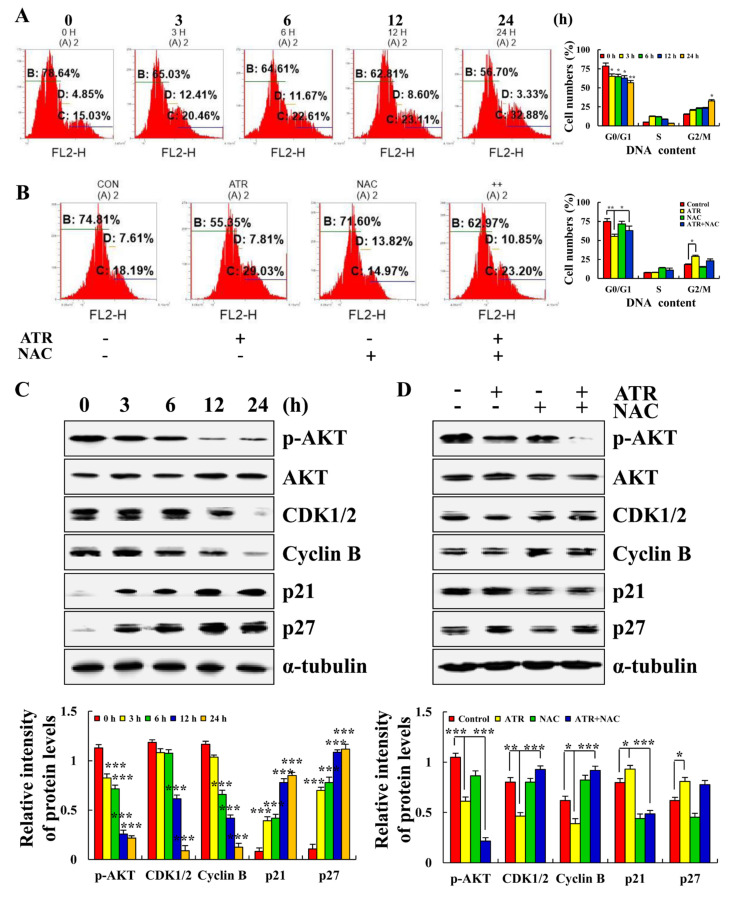
Effects of ATR on cell cycle arrest of A549 cells. A549 cells treated with 38 µM ATR for 3, 6, 12, and 24 h. (**A**) RNase and PI staining, flow cytometry analysis of cell cycle. (**C**) Protein expression levels of p-AKT, CDK1/2, Cyclin B, p21, and p27 detected by Western blot analysis. * *p* < 0.05, ** *p* < 0.01 and *** *p* < 0.001 vs. 0 h. A549 cells treated with 38 µM ATR and/or 10 mM NAC for 24 h. (**B**) Rnase and PI staining, flow cytometry analysis of cell cycle. (**D**) Protein expression levels of p-AKT, CDK1/2, Cyclin B, p21, and p27 detected by Western blot analysis. α-Tubulin used as internal reference. * *p* < 0.05, ** *p* < 0.01 and *** *p* < 0.001 vs. control or ATR + NAC groups.

ATR induced ROS accumulation and then mediates AKT signaling pathway to arrest A549 cell cycle in G2/M phase. Compared with the untreated groups, the number of cells in G0/G1 phase decreased from 78.64% to 56.70% in the ATR-treated group, and the number of cells in G2/M phase increased from 15.03% to 32.88% (Figure 5A). In addition, in order to explore the relationship between ATR-induced G2/M phase cell arrest and ROS accumulation, NAC was added for pretreatment. NAC inhibited the increase in ATR-induced G2/M phase cells (Figure 5B). The expression of cell cycle-related proteins was further detected. The protein expression levels of p21 and p27 increased, whereas the protein expression levels of p-AKT, CDK1/2, and cyclin B decreased (Figure 5C). NAC also inhibited ATR-induced protein expression (Figure 5D).

### 2.6. ATR Inhibits A549 Cell Migration

**Figure 6:**
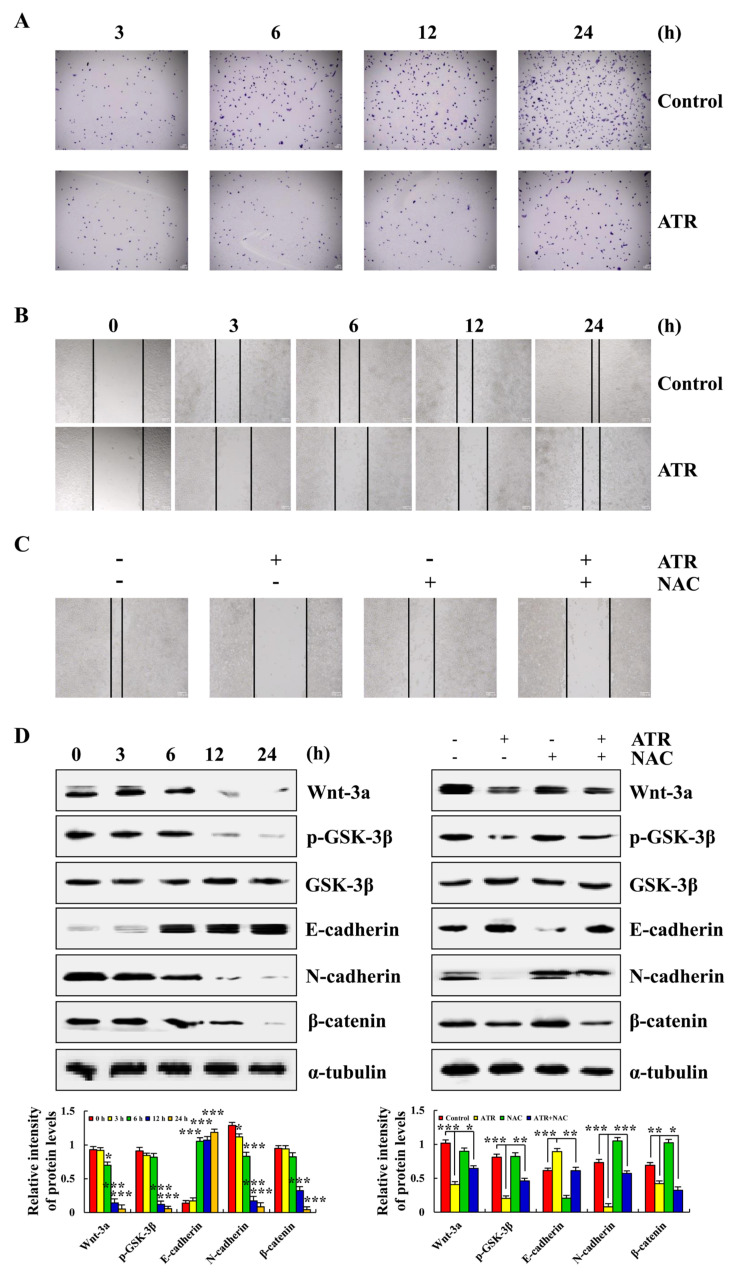
Effects of ATR on migration of A549 cells. A549 cells treated with 38 µM ATR for 3, 6, 12, and 24 h, followed by fluorescence microscopy (original magnification, 200×). (**A**) Transwell assay analysis for cell migration. (**B**) Wound-healing assay analysis of cell migration rate. A549 cells treated with 38 µM ATR and/or 10 mM NAC for 24 h. (**C**) Wound healing assay analysis of cell migration rate. (**D**) Protein expression levels of Wnt-3a, p-GSK-3β, E-cadherin, N-cadherin, and β-catenin were detected by Western blot analysis. α-Tubulin used as the internal reference. * *p* < 0.05, ** *p* < 0.01 and *** *p* < 0.001 vs. 0 h, control, or ATR + NAC groups.

ATR induced ROS accumulation and mediated the Wnt signaling pathway to inhibit A549 cell migration. As shown in Figure 6A,B, compared with the untreated group, the cell migration and cell migrate rate of A549 cells after 24 h of ATR treatment were significantly reduced. In addition, in order to explore the relationship between the ATR inhibition of cell migration and ROS accumulation, NAC was added for pretreatment. NAC accelerated the cell migration rate compared with the ATR treatment groups (Figure 6C). The expression of migration-related proteins was further detected. The expression levels of Wnt-3a, p-GSK-3β, N-cadherin, and β-catenin decreased, whereas the expression level of E-cadherin increased, and NAC also inhibited ATR-induced protein expression (Figure 6D).

## 3. Discussion

ATR was mainly derived from the dried roots of Compositae plant *Atractylodes lancea*. ATR presented pharmacological activities such as lowering blood sugar, and anti-inflammation and antitumor effects [25,26,27,28,29,30,31]. A study showed that, after treatment with ATR for 24, 48, and 72 h, IC_50_ values of HuCCT1 cholangiocarcinoma cells were 240, 195, and 162 µM, respectively [32]. After treatment with ATR for 24 h, the IC_50_ value of Hun 7 liver cancer cells was 22.36 µM [33]. In addition, 80 mg/L ATR inhibited the proliferation of LS174T colon cancer cells by nearly 100% [31]. The above studies proved that ATR has a good inhibitory effect on the growth of some tumor cells, but its mechanism of action on lung cancer has not been reported. Although one study showed that ATR had no toxic effect on A549 cells, we repeatedly analyzed the survival rate of lung cancer cells treated with ATR [34]. Results showed that ATR could inhibit the proliferation of lung cancer cells A549, NCI-H23, NCI-H460, and HCC827, and the effect was better than that of 5-FU, especially for A549 cells. Therefore, we conducted a series of follow-up studies to analyze the effects of ATR on the apoptosis, cell cycle, and migration of A549 cells. Results showed that ATR could kill A549 cells, induce apoptosis and cell cycle arrest, and inhibit cell migration, showing good anticancer effects.

Apoptosis affects physiological processes such as growth, differentiation, and immune regulation, and is closely related to pathological processes such as cancer, which is one of the most active fields in biological research [35]. MAPK, STAT3, NF-κB, and other signaling pathways can activate the core apoptotic enzyme, namely, caspase, thereby promoting the cleavage of related proteins and completing the apoptotic process [35,36]. Upstream of caspase, there are various Bcl-2 family members that play a role in determining cell survival by influencing the formation or degradation of caspase and promoting or inhibiting the apoptotic process [37]. In addition, the MMP of apoptotic cells changes, and Bcl-2 family members distributed on the mitochondrial membrane promote the release of cytochrome c [38]. Cytochrome c released by mitochondria also activates caspase [39]. The results of this study showed that ATR induces the release of cytochrome c by reducing MMP, and plays a role in the apoptosis of A549 cells. To further investigate whether ATR induces apoptosis of A549 cells by regulating the MAPK, STAT3 and NF-κB signaling pathways, we analyzed the expression levels of related proteins by Western blot. Results showed that ATR decreased the expression of Bcl-2, p-ERK, p-STAT3 and NF-κB-p65, and increased the expression of Bad, cytochrome c, cle-caspase-3, cle-PARP, p-JNK, p-p38, and p-IκBα during the induction of apoptosis of A549 cells. In addition, similar to previous findings, the STAT3 signaling pathway is regulated by the MAPK signaling pathway [40,41]. These results confirm our conjecture and the specific molecular mechanism of ATR inducing the apoptosis of A549 cells.

During electron transfer in the mitochondrial respiratory chain, most electrons are transferred to O_2_ by oxidase through the respiratory chain and generate H_2_O. However, a small portion of single electrons leak out from the respiratory chain and generate superoxide anion (•O_2_^−^) [42]. •O_2_^−^ is decomposed by the superoxide dismutase of mitochondria into hydrogen peroxide, which eventually generates hydroxyl radical (•OH), and these are the main components of ROS [43,44,45]. ROS play a role in apoptosis by changing the redox state of cells and regulating signal transduction pathways [46]. Therefore, the ROS levels of A549 cells treated with ATR were measured in this study. Results showed that the increase in ATR-induced ROS induced changes in the MAPK, STAT3, and NF-κB signaling pathways. This study confirmed that ATR induces the apoptosis of A549 cells by promoting ROS accumulation.

Each stage of the cell cycle is tightly regulated, and checkpoints exist to detect potential DNA damage and allow for repair before the cell divides [47]. If the damage cannot be repaired, the cell dies. Therefore, many drugs work by arresting the cell cycle of cancer cells. A previous study showed that LS174T colon cancer cells treated with 40 mg/L ATR increased the proportion of G0/G1 phase cells [31]. Further detection at this concentration showed that ATR treatment for 24 and 48 h significantly reduced the secretion of interleukin 6 (IL-6) and IL-7 in LS174T cells, which inhibited cell growth [31]. Another study showed that an increase in ATR concentration in Huh7 hepatoma cells led to a gradual increase in the number of cells in the G1 phase, and a gradual decrease in the number of cells in the G2 phase [33]. We examined ATR-treated A549 cells and found the opposite results. ATR led to a gradual decrease in the number of cells in the G0/G1 phase, and a gradual increase in the number of cells in G2/M phase, suggesting that ATR blocked the A549 cell cycle in the G2/M phase. Further investigation into the molecular mechanism revealed that the cell cycle arrest of A549 cells was regulated by the ATR-induced ROS-mediated AKT signaling pathway.

Cell migration is involved in embryonic development, wound healing, inflammatory response, and cancer metastasis [48]. Some studies showed that ATR inhibits the migration and invasion of HuCCT1 cells and Huh7 cells in a concentration-dependent manner [25,26]. In addition, ATR inhibits the colony-forming ability and weakens the wound healing ability of CL-6 cholangiocarcinoma cells [49]. These studies showed that ATR can inhibit the migration of tumor cells, but did not reveal the specific mechanism of action. Therefore, in this study, we demonstrated the migration inhibition effect of ATR on A549 cells by the Transwell and cell scratch assays. On the molecular level, expression levels of proteins related to the canonical Wnt signaling pathway were detected, and ATR inhibited the expression of Wnt-3a and β-catenin proteins, ultimately inhibiting the migration of A549 cells. In addition, N-cadherin and E-cadherin both belong to classical cadherin, and the two genes are located on chromosomes 18 and 16, respectively [50,51]. The mechanisms of N-cadherin and E-cadherin in mediating cell adhesion are the same, but the roles of N-cadherin and E-cadherin in tumor metastasis are the opposite [52]. When normal cells turn malignant, E-cadherin turns into N-cadherin [53]. Our results showed that, after A549 cells had been treated with ATR, the expression of N-cadherin protein decreased, while the expression of e-cadherin protein increased, which further indicated that ATR could inhibit the migration of A549 cells.

**Figure 7:**
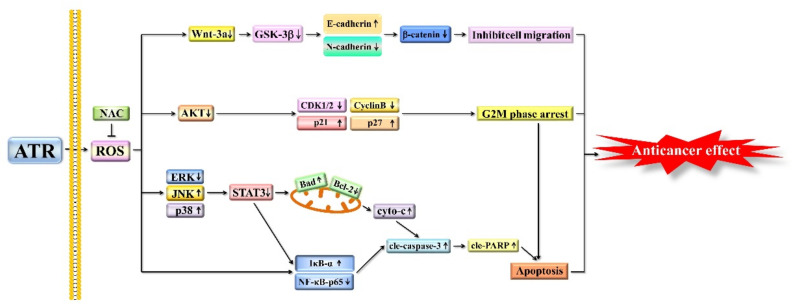
Schematic diagram of anticancer role of ATR in A549 cells. Molecular mechanism of ATR anticancer activity related to ROS production, and MAPK, STAT3, NF-κB, AKT, and Wnt signaling pathways.

In conclusion, ATR induces the apoptosis, G2/M phase arrest, and migration inhibition of A549 cells through ROS accumulation (Figure 7). Therefore, ATR may be a candidate drug for the treatment of lung cancer.

## 4. Materials and Methods

### 4.1. Chemicals

ATR (purity > 98%) (Herbpurify; Chengdu, China) or 5-FU (Med Chem Express, Princeton, NJ, USA) was dissolved in dimethyl sulfoxide (DMSO) (Solarbio, Beijing, China) and stored at −20 °C. The final concentration of DMSO was less than 0.5%, which could rule out the cytotoxic effect of the solvent on cells. The chemical concentration was diluted with cell culture medium before use.

### 4.2. Cell Lines and Culture

Lung cancer cells (A549, NCI-H23, NCI-H460, and HCC827) and normal human cells (IMR-90, GES-1, THLE-2, and 293T) were used in the study. Lung-cancer and IMR-90 cells were purchased from American Type Culture Collection (Manassas, VA, USA). GES-1 and 293T cells were purchased from Sage Biotechnology Co. (Shanghai, China). THLE-2 cells were purchased from Otwo Biotech (Shenzhen, China). All cells were grown in Dulbecco’s Modified Eagles Medium (DMEM) (Gibco, Waltham, MA, USA). The cell culture medium was supplemented with 10% fetal bovine serum (FBS) (Gibco, Waltham, MA, USA) and 1% penicillin/streptomycin (Solarbio, Beijing, China). Cells were grown in an incubator at 37 °C and 5% CO_2_.

### 4.3. Cytotoxic Assay

Cell viability was detected using Cell Counting Kit-8 (CCK-8) (Solarbio, Beijing, China). Briefly, all cells (1 × 10^4^ cells/well) were cultured in 96-well plates for 24 h. Cells were treated with ATR and 5-FU at 20, 40, 60, 80, and 100 µM. A total of 10 μL CCK-8 was added to each well and incubated for 3 h in the dark. Absorbance was measured at 450 nm using a multifunctional microplate reader (Tecan, Mannedorf, Switzerland). IC_50_ values were calculated using GraphPad Prism software. In addition, all cells were treated with IC_50_ values of ATR and 5-FU for 6, 12, 18, 24, and 30 h to analyze the cell survival rate.

### 4.4. Cell Apoptosis Assay

The apoptosis of A549 cells was detected using the Annexin V-FITC/PI Apoptosis Kit (4A Biotech, Beijing, China). Briefly, A549 cells (1 × 10^5^ cells/well) were cultured in 6-well plates for 24 h. Cells were treated with 38 µM (the IC_50_ value of A549 cells) ATR and 38 µM 5-FU for 3, 6, 12, and 24 h. Collected cells were evenly mixed with 200 µL 1× binding buffer, 3 µL Annexin V-FITC, and 5 µL PI, and incubated for 15 min in the dark. Cell morphology was observed by fluorescence microscopy (MSHOT, Guangzhou, China). In addition, ATR-treated A549 cells were mixed with 100 µL 1× binding buffer, followed by the addition of 4 µL Annexin V-FITC and 3 µL PI and incubation for 5 min away from light. The rate of cell apoptosis was detected by flow cytometry (Sysmex Co., Kobe, Japan).

### 4.5. MMP Assay

The MMP of A549 cells was detected using the JC-1 MMP Detection Kit (Solarbio, Beijing, China). Briefly, A549 cells (1 × 10^5^ cells/well) were cultured in 3.5 cm cell culture dishes for 24 h. Cells were treated with 38 µM ATR for 3, 6, 12, and 24 h. Collected cells were stained with 1 mL JC-1 staining solution for 30 min, followed by the addition of 1 mL 1× JC-1 binding buffer and 300 µL phosphate-buffered saline (PBS). MMP was analyzed using flow cytometry.

### 4.6. Nuclear Protein Extraction

Nuclear protein was extracted from A549 cells using nuclear protein extraction kits (Solarbio, Beijing, China). Briefly, A549 cells (6 × 10^5^ cells/well) were cultured in 6 cm cell culture dishes for 24 h. Cells were treated with 38 µM ATR for 3, 6, 12, and 24 h. Collected cells were centrifuged at 500× *g* for 3 min, followed by the addition of 200 μL cytoplasmic extraction reagent and incubation on ice for 10 min. The cell solution was centrifuged at 15,000× *g* for 10 min, and the supernatant was cytoplasmic protein. Then, 80 μL nuclear protein extraction reagent was added to the cell precipitate, followed by centrifugation at 14,000× *g* for 10 min; the supernatant was nuclear protein.

### 4.7. Western Blot Analysis

Protein expression was detected by Western blot analysis. Briefly, A549 cells (6 × 10^5^ cells/well) were cultured in 6 cm cell culture dishes for 24 h. Cells were treated with 38 µM ATR for 3, 6, 12, and 24 h. Proteins were separated on 12% SDS-PAGE electrophoresis gels and electrotransferred to nitrocellulose membranes. After adding 5% skim milk to avoid nonspecific binding, the membrane was incubated with primary antibodies (Santa Cruz Biotechnology, Dallas, TX, USA) at 4 °C overnight. The primary antibodies used were Bad (25 kDa), Bcl-2 (26 kDa), cyto-c (15 kDa), cle-caspase-3 (17 kDa), cle-PARP (89 kDa), α-tubulin (55 kDa), p-ERK (42 kDa), ERK (42 kDa), p-JNK (46 kDa), JNK (46 kDa), p-p38 (38 kDa), p38 (38 kDa), p-STAT3 (91/86 kDa), STAT3 (91/86 kDa), NF-κB-p65 (65 kDa), IκBα (35–41 kDa), p-IκBα (41 kDa), Lamin B1 (67 kDa), p-AKT (56 kDa), AKT (56 kDa), CDK1/2 (33 kDa), Cyclin B (60 kDa), p21 (21 kDa), p27 (27 kDa), Wnt-3a (65 kDa), p-GSK-3β (47 kDa), GSK-3β (47 kDa), E-cadherin (120 kDa), N-cadherin (130 kDa) and β-catenin (92 kDa). Next, the membrane was incubated with secondary antibodies (ZSGB-bio, Beijing, China) for 2 h. Lastly, proteins were detected with enhanced chemiluminescence (Tanon, Shanghai, China). Images were exposed with a multifunction imager (Analytik Jena AG, Jena, Germany) and analyzed with ImageJ software.

### 4.8. ROS Accumulation Analysis

The ROS levels of A549 cells were detected using ROS detection kits (Beyotime Institute Biotechnology, Shanghai, China). Briefly, A549 cells (1 × 10^5^ cells/well) were cultured in 3.5 cm cell culture dishes for 24 h and treated with 38 µM ATR for 3, 6, 12, and 24 h. Collected cells were stained with 10 µL 2’,7’-dichlorofluorescein diacetate (DCFH-DA) and incubated for 30 min in the dark, followed by the addition of 300 µL PBS. ROS accumulation was detected by flow cytometry. In addition, A549 cells had been pretreated with NAC (Beyotime Institute Biotechnology) before being treated with ATR and tested again.

### 4.9. Cell Cycle Analysis

The cell cycle of A549 cells was detected using DNA quantification kits (Solarbio, Beijing, China). Briefly, A549 cells (1 × 10^5^ cells/well) were cultured in 3.5 cm cell culture dishes for 24 h. Cells were treated with 38 µM ATR for 3, 6, 12, and 24 h. The collected cells were fixed in 70% alcohol at 4 °C overnight. Then 100 µL RNase was added and incubated for 30 min at 37 °C, followed by the addition of 400 µL PI and staining at 4 °C for 30 min in the dark. The number of cells at different stages was detected by flow cytometry. Then the A549 cells were pretreated with NAC before being treated with ATR and tested again.

### 4.10. Cell Migration Analysis

Cell migration was detected by wound-healing assay. Briefly, A549 cells (1 × 10^5^ cells/well) were cultured in 6-well plates for 24 h and then vertically scratched. After 2 h culture in serum-free DMEM, cells were treated with 38 µM ATR for 3, 6, 12, and 24 h. Cell migration was observed by fluorescence microscope. In addition, A549 cells were pretreated with NAC before being treated with ATR and tested again. Cell migration was detected by Transwell assay. Briefly, a serum-free suspension of A549 cells (1 × 10^5^ cells/well) treated with 38 µM ATR was cultured in the upper chamber. DMEM containing 20% FBS was added to the lower chamber. A549 cells were stained with 1% crystal violet solution, and cell migration was observed at 3, 6, 12, and 24 h.

### 4.11. Statistical Analyses

The experiment was repeated three times, and data are expressed as mean ± standard deviation. GraphPad Prism software was used to calculate IC_50_, and SPSS 21.0 software was used for Tukey’s post hoc test. *p* < 0.05 was considered to be statistically significant.

## Figures and Tables

**Table 1:** IC_50_ values of ATR and 5-FU in lung cancer cells.

Cell Line	5-FU (µM)	ATR (µM)
A549	64.24 ± 2.58	37.92 ± 1.59
NCI-N23	90.21 ± 1.44	76.88 ± 2.21
NCI-H460	108.36 ± 2.59	63.27 ± 1.48
HCC827	99.62 ± 2.41	61.05 ± 1.66

IC_50_ values calculated using GraphPad Prism software.

## Data Availability

Not applicable.

## Sample Availability

Samples of the compounds are not available from the authors.
